# *Caenorhabditis elegans* BUB-3 and SAN-1/MAD3 Spindle Assembly Checkpoint Components Are Required for Genome Stability in Response to Treatment with Ionizing Radiation

**DOI:** 10.1534/g3.117.1122

**Published:** 2017-10-18

**Authors:** Simone Bertolini, Bin Wang, Bettina Meier, Ye Hong, Anton Gartner

**Affiliations:** Centre for Gene Regulation and Expression, School of Life Sciences, University of Dundee, DD1 5EH, UK

**Keywords:** ionizing radiation, spindle assembly checkpoint, BUB-3, SAN-1/MAD-3, DNA damage response

## Abstract

Relatively little is known about the cross-talk between the spindle assembly checkpoint and the DNA damage response, especially in multicellular organisms. We performed a *Caenorhabditis elegans* forward genetic screen to uncover new genes involved in the repair of DNA damage induced by ionizing radiation. We isolated a mutation, *gt2000*, which confers hypersensitivity to ionizing radiation and showed that *gt2000* introduces a premature stop in *bub-3*. BUB-3 is a key component of the spindle assembly checkpoint. We provide evidence that BUB-3 acts during development and in the germline; irradiated *bub-3*(*gt2000*) larvae are developmentally retarded and form abnormal vulvae. Moreover, *bub-3*(*gt2000*) embryos sired from irradiated worms show increased levels of lethality. Both *bub-3* and *san-1* (the *C. elegans* homolog of MAD3) deletion alleles confer hypersensitivity to ionizing radiation, consistent with the notion that the spindle assembly checkpoint pathway is required for the DNA damage response. *bub-3*(*gt2000*) is moderately sensitive to the cross-linking drug cisplatin but not to ultraviolet light or methyl methanesulfonate. This is consistent with a role in dealing with DNA double-strand breaks and not with base damage. Double mutant analysis revealed that *bub-3* does not act within any of the three major pathways involved in the repair of double-strand breaks. Finally, the *cdc-20* gain-of-function mutant *cdc-20/fzy-1*(*av15*), which is refractory to the cell cycle delay conferred by the spindle checkpoint, showed phenotypes similar to *bub-3* and *san-1* mutants. We speculate that BUB-3 is involved in the DNA damage response through regulation of cell cycle timing.

Faithful DNA replication and chromosome segregation are essential for maintaining genome integrity. To ensure the high fidelity of these processes, checkpoint mechanisms have evolved to delay cell cycle progression when DNA damage is sensed or chromosome alignment is incomplete. The DNA damage checkpoint senses DNA lesions using the ATM and ATR apical sensors to effect transient cell cycle arrest and efficient DNA repair. By contrast, the spindle assembly checkpoint (SAC) was classically implicated in delaying anaphase onset until all mitotic chromosomes are aligned at the mitotic spindle. Failure to do so can lead to chromosome mis-segregation and ensuing aneuploidy. It was established that the SAC delays progression to anaphase when chromosomes are not attached to the kinetochore by inhibiting the Cdc20/FZY-1 activator of the anaphase promoting complex (APC) (Hwang *et al.* 1998). The APC is an E3 ubiquitin ligase that triggers anaphase by inducing the degradation of cyclin B and securin. The latter protein binds to and thereby inhibits separase, a protease that allows for the separation of chromatids by cohesin cleavage. Current models posit that three conserved SAC proteins (Mad2, Bub3, and Mad3/BubR1) interact with each other to generate the mitotic checkpoint complex (MCC) that is responsible for Cdc20/FZY-1 inhibition (Musacchio and Salmon 2007; Lara-Gonzalez *et al.* 2012; Primorac and Musacchio 2013). The SAC protein Mad2 adopts two native conformations, namely the “open” (O-Mad2) and “closed” (C-Mad2) states. According to the “Mad2 template model” (De Antoni *et al.* 2005), Mad2 exists as the inactive diffusible O-Mad2 conformer when kinetochores are correctly attached to the spindle. In presence of unbound kinetochores, a fraction of Mad2 proteins adopt the C-Mad2 active state to form a tetrameric 2:2 complex with Mad1 on the unattached kinetochores. Mad1-bound C-Mad2 recruits O-Mad2 at the unattached kinetochore to facilitate the interaction between O-Mad2 and Cdc20/FZY-1. Upon binding to Cdc20/FZY-1, O-Mad2 switches conformation to the C-Mad2 state. The C-Mad2:Cdc20 complex is then released to the cytoplasm and leads to the inhibition of the APC (Musacchio and Salmon 2007). In parallel to Mad2 activation, Bub3 and Mad3/BubR1 form a dimer that binds to C-Mad2:Cdc20, thereby assembling the MCC (Essex *et al.* 2009). The active MCC persists until all chromosomes have achieved bipolar attachment to the mitotic spindle. Once this is achieved, the MCC is disassembled and Cdc20/FZY-1 promotes anaphase by activating the APC. In addition to its function in checkpoint signaling, Bub3 was recently shown to promote metaphase-to-anaphase transition in the absence of spindle perturbation (Kim *et al.* 2015).

Although the SAC is active at low levels in unperturbed S-phase to ensure timely onset of mitosis (Magiera *et al.* 2014), it is not essential for the growth of haploid budding yeast cells in the absence of spindle perturbation. Components of the SAC were initially found in genetic screens for mutants that bypass the mitotic cell cycle arrest phenotype conferred by the microtubule poisons nocodazole and benomyl (Hoyt *et al.* 1991; Li and Murray 1991). In contrast to haploid yeast, most homologs of the SAC genes are required for viability in animals even in the absence of spindle damage (Gorbsky *et al.* 1998; Wild *et al.* 2016). This is thought to be due to the role of the SAC in delaying anaphase onset (Wild *et al.* 2016). Indeed, the delay of anaphase onset by the SAC is also required for ordered segregation of chromosomes during the first meiotic division in budding yeast (Shonn *et al.* 2000). In mouse, MAD2 deficiency does not allow embryos to develop beyond the E6.5 stage (Dobles *et al.* 2000). In *Caenorhabditis elegans*, depletion of BUB-1 by RNAi causes high levels of embryonic lethality (Tarailo *et al.* 2007). Loss-of-function *mdf-1*^MAD-1^ mutants display severe defects during larval development that prevent strain propagation (Kitagawa and Rose 1999; Stein *et al.* 2007). Similarly, loss of MAD-2 results in low brood size, reduced progeny viability, and high frequency of larval defects (Kitagawa and Rose 1999; Stein *et al.* 2007). By contrast, BUB-3 and MAD-3 appear to be dispensable for survival under physiological conditions in *C. elegans* (Nystul *et al.* 2003; Tarailo *et al.* 2007; Hajeri *et al.* 2008).

Several lines of evidence indicate that the SAC and the DNA damage response (DDR) have overlapping functions. Although the SAC was initially believed not to participate in the DDR (Hoyt *et al.* 1991; Hardwick *et al.* 1999), it was later shown that Mad1p and Mad2p contribute to the preanaphase arrest induced by DNA replication defects and the DNA-damaging agent methyl methanesulfonate (MMS) in budding yeast (Garber and Rine 2002; Palou *et al.* 2017). It was hypothesized that damaged centromeric DNA disrupts the structure of kinetochores and, as a result, altered kinetochores elicit SAC-dependent cell cycle arrest. However, the role of kinetochores in DNA damage-induced cell cycle arrest has been called into question, as mutants that lack kinetochores are still capable of sustaining a durable arrest in the presence of DNA damage (Kim and Burke 2008). Nevertheless, a clear role for the centromere in the DDR has been established in *S. cerevisiae* when a double-strand break (DSB) is induced within a 100,000 bp distance of the centromere (Dotiwala *et al.* 2010). The full cell cycle arrest conferred by this persistent DSB is dependent on the SAC and the DNA damage checkpoint pathways and requires histone modifications at centromeric DNA (Dotiwala *et al.* 2010). It was suggested that a DSB close to a centromere leads to altered chromatin conformation that triggers kinetochore dysfunction recognized by the SAC (Dotiwala *et al.* 2010). Another role for SAC proteins appears to be to confer efficient cell cycle arrest when single-stranded DNA is enriched at subtelomeric regions upon depletion of the nonhomologous end-joining DNA repair factor yKu70∆ in *S. cerevisiae* (Maringele and Lydall 2002). It was suggested that chromosome fusions occurring in yKu70∆ mutants lead to the formation of dicentric chromosomes, which have previously been shown to trigger the SAC (Neff and Burke 1992). Cross-talk between the DNA damage checkpoint and the SAC appears to be conserved from yeast to humans. p53-deficient cancer cells treated with DNA polymerase inhibitor aphidicolin elicit a BubR1-dependent metaphase arrest (Nitta *et al.* 2004). Similar observations were obtained from a study in murine fibroblasts (Fang *et al.* 2006). Interestingly, some *C. elegans sac* mutants show persistent DNA double-strand breaks upon exposure to ionizing radiation (IR) and upon hydroxyurea treatment, which blocks DNA replication (Lawrence *et al.* 2015). These DNA-damaging agents induce MAD-2 to colocalize with damaged DNA at the nuclear periphery of proliferating germ cells in interphase. Peripheral localization of MAD-2 is dependent on the DDR kinase ATR. These results are in line with the DNA damage-induced cell cycle arrest phenotype being alleviated in *mad-2* mutants and MAD-2 possibly also playing a direct part in DSB repair at the nuclear periphery (Lawrence *et al.* 2015).

In this study, we isolated a *C. elegans* strain that carries a mutation in the SAC gene *bub-3* using a forward genetic approach. *bub-3* mutants are hypersensitive when exposed to IR and to the DNA cross-linking agent cisplatin. Epistasis analysis suggests that *bub-3* acts independently of the major DNA repair pathways involved in DNA double-strand break repair. Moreover, the characterization of a *cdc-20* gain-of-function allele, *fzy-1*(*av15*), suggests that SAC proteins might have a role in regulating cell cycle timing in response to DNA damage.

## Materials and Methods

### C. elegans strains and maintenance

*C. elegans* strains were maintained at 20° on *Escherichia coli* OP-50 seeded nematode growth media (NGM) agar plates as described previously (Brenner 1974). The N2 Bristol reference line TG1813 is used in the Gartner laboratory as the wild-type reference strain. All mutant strains were outcrossed six times to TG1813 except *bub-3* (*gt2000*), which was outcrossed three times to TG2435. Strains used in this paper are: TG1813 N2 Bristol, TG2435 *vtIs1*[*pdat-1*::*gfp*; *rol-6*] *V*, CB4856 Hawaii, TG3796 *bub-3*(*gt2000*) *II*, RB1391 *san-1*(*ok1580*) *I*, VC2773 *bub-3*(*ok3437*) *II*, TG1660 *xpf-1*(*tm2842*) *II*, DW102 *brc-1*(*tm1145*) *III*, RB873 *lig-4*(*ok716*) *III*, TG2534 *polq-1*(*tm2026*) *III*, RB2422 *polh-1*(*ok3317*) *III*, TG1540 *gen-1*(*tm2940*) *III*, TG3899 *bub-3*(*gt2000*) *II*; *brc-1*(*tm1145*) *III*, TG3870 *bub-3*(*gt2000*) *II*; *polq-1*(*tm2026*) *III*, TG3900 *bub-3*(*gt2000*) *II*; *lig-4*(*ok716*) *III*, TG4071 *fzy-1*(*av15*) *II*, TG4085 *fzy-1*(*av15*) *bub-3*(*gt2000*) *II*, TG4092 *bub-3(knu207*[*Pbub-3*::*eGFP*::*bub-3*::*3′UTRbub-3*)], TG4193 *bub-3(knu207*[*Pbub-3*::*eGFP*::*bub-3*::*3′UTRbub-3*)]; *odIs57*[*Ppie-1*::*mCherry*::*histoneH2B + unc-119*(*+*)]; *unc-119*(*ed3*), TG4196 *odIs57*[*Ppie-1*::*mCherry*::*histoneH2B + unc-119*(*+*)]; *ItIs38*[*pie-1*::*GFP*::*PH*(*PLC1delta1*) *+ unc-119*(*+*)]; *unc-119*(*ed3*), TG4197 *odIs57*[*Ppie-1*::*mCherry*::*histoneH2B + unc-119*(*+*)]; *ItIs38*[*pie-1*::*GFP*::*PH*(*PLC1delta1*) *+ unc-119*(*+*)]; *unc-119*(*ed3*); *bub-3*(*gt2000*) *II*. The *bub-3(knu207*[*Pbub-3*::*eGFP*::*bub-3*::*3′UTRbub-3*)] eGFP insertion was generated by Knudra (http://www.knudra.com/) following the procedures described in Dickinson *et al.* (2015). Exact details are available upon request.

### Mutagenesis screen and mutation identification

Mutagenesis and screening procedures were performed as described in González-Huici *et al.* (2017). Single nucleotide polymorphism (SNP) mapping was performed according to the protocol described in Davis *et al.* (2005). For whole-genome sequencing, genomic DNA was extracted and purified using a ChargeSwitch gDNA mini tissue kit (Invitrogen) and sent to GenePool (http://genepool.bio.ed.ac.uk/) for Illumina (Solexa) sequencing. Paired-end sequencing was set to achieve 24× coverage (100 bp paired-end reads for a total of 24,000,000 reads). Quality of the reads was checked using FastQC. Reads were then aligned to the *C. elegans* reference genome (WBcel235.74) using BWA-MEM. Variants in the strains TG1813 and TG2435 were called using the software SAMtools and Bfctools. Heterozygous variants and variants that were not unique to the mutant strain were filtered out. We then extracted homozygous variants within the 900 kb region determined by SNP mapping. Homozygous unique variants were then ranked based on the severity of the predicted effect on the genome. The *gt2000* mutation was supported by 18 sequence reads including reads from both directions, confirmed visually using the Integrative Genomics Viewer software.

### Sensitivity assays

For the L1 sensitivity assay, gravid adults were bleached and eggs were incubated at 20° under shaking for at least 13 hr to obtain synchronized populations of L1 larvae. Larvae were plated on seeded NGM plates and irradiated at the indicated doses using a ^137^Cs source (IBL 437C; CIS Bio International). Animals that developed into L4 larvae within 49 hr post irradiation were scored, as well as the total number of plated larvae. Ruptured worms were scored 72 hr post irradiation as percentage of total number of plated worms. For IR and ultraviolet (UV) treatments of young adults, animals were irradiated at the indicated doses. After 24 hr one worm was singled out on a plate, to allow for egg-laying for 12 hr. The adult was then removed and the number of laid eggs was scored. The number of dead (unhatched eggs) embryos was scored 24 hr after removal of the adult. A minimum of six plates per condition were analyzed. For genotoxin treatment, young adults were incubated in liquid solution [M9 buffer (3 g/L KH2PO4, 6 g/L Na2HPO4, 5 g/L NaCl, 1 mM MgSO4) + OP50 + genotoxins at indicated concentration] at 20° under shaking for 16 hr. After incubation, worms were washed with fresh M9 buffer and transferred onto seeded NGM plates for 24 hr to recover before being transferred again onto freshly seeded NGM plates for 6 hr to lay eggs (three worms per plate for a total of three plates per condition). The number of laid eggs was scored immediately after removal of adults. Dead eggs were scored 24 hr after removal of adults. For irradiation of late-stage embryos, we followed the protocol described in Clejan *et al.* (2006).

### DAPI- and immunostaining

DAPI staining of oocytes and RAD-51 immunostaining was performed as described in González-Huici *et al.* (2017). For DAPI staining of whole germlines, we used a procedure described in Craig *et al.* (2012). For phosphoCDK-1^Tyr15^ immunostaining, we followed the protocol described in Moser *et al.* (2009). Anti-RAD-51 antibody was diluted 1:800, whereas anti-phosphoCDK-1^Tyr15^ antibody was diluted to 1:100. Secondary antibody (donkey anti-rabbit conjugated with Alexa Fluor 568; Thermo Fisher Scientific) was diluted to 1:750 and to 1:1000 for RAD-51 and phosphoCDK-1^Tyr15^ immunostaining, respectively. A DeltaVision wide-field microscope with Coolsnap HQ camera and softWoRx software was used to acquire fluorescence images. To analyze and process images, we used softWoRx and Adobe Photoshop software.

### Time-lapse live embryo imaging

For time-lapse imaging of live embryos, we followed an imaging procedure as described in Sonneville *et al.* (2015). Young adults were irradiated as described in the embryonic lethality assay. One-cell embryos were dissected in M9 buffer 24 hr post irradiation and immediately mounted on 2% agarose pads. Images were acquired every 10 sec using a spinning-disk confocal microscope (IX81; Olympus) with spinning-disk head (CSU-X1; Yokogawa Electric Corporation) and MetaMorph software (Molecular Devices). For image processing, we used the ImageJ software.

### Data availability statement

*bub-3* and *mad-3* mutant strains were sent to the *Caenorhabditis* Genetics Center (GCG) *C. elegans* strain collection. Other strains are available upon request.

## Results

**Figure 1:**
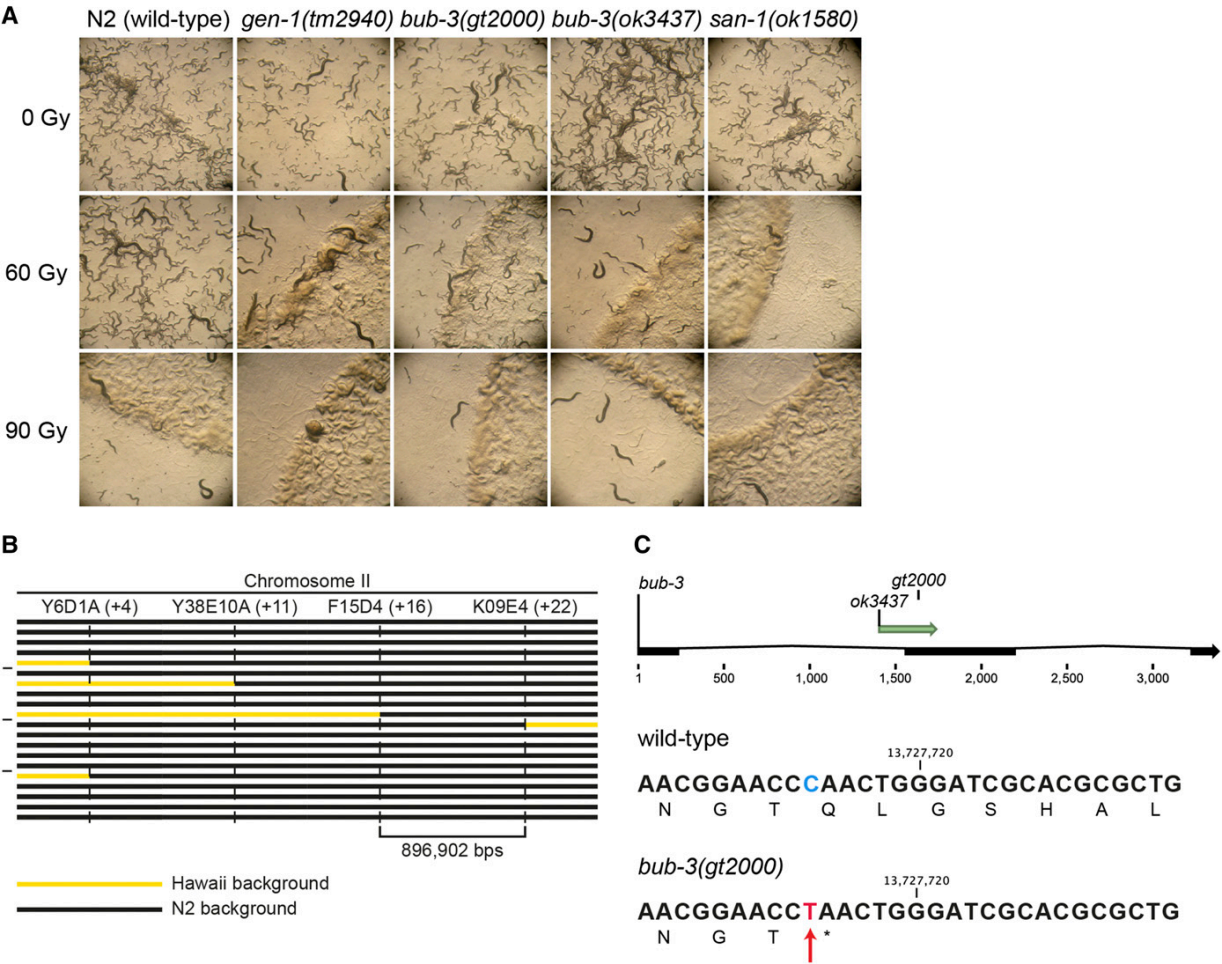
*sac* mutants are hypersensitive to IR. (A) Representative images of NGM plates 5 d after irradiation of L1 larvae. Wild-type animals irradiated with 60 Gy propagated normally, whereas *sac* mutants *bub-3* and *san-1* showed impaired growth similar to *gen-1*(*tm2940*). (B) Schematic of the SNP mapping. The horizontal bars represent the right arm of chromosome II in 20 IR-sensitive F2 lines derived from a cross between CB4856 (Hawaii) and the IR-sensitive mutant. Black segments identify genomic regions that contain N2 SNPs. Yellow segments correspond to genomic regions that contain Hawaiian SNPs. Vertical dashed lines show the genetic position of the indicated SNPs. *gt2000* was mapped between F14D4 and K09E4 on the physical map (top line) to a ∼900 kb region that shows only N2 SNPs in all F2 lines. (C) Schematic of the exon-intron structure of *bub-3* with the location of the *ok3437* deletion and the *gt2000* point mutation indicated (top panel). DNA sequence surrounding the *gt2000* allele in wild type and *bub-3*(*gt2000*) including the corresponding amino acid sequence is shown. The C > T substitution in *bub-3*(*gt2000*), which causes a premature stop codon, is indicated by a red arrow.

To uncover new genes involved in the DDR, we performed an unbiased forward genetic screen. Upon ethyl methanesulfonate (EMS) mutagenesis of P0 wild-type (N2) individuals, F2 animals were singled in 96-well plates (González-Huici *et al.* 2017). Progeny of singled L4 stages animals was split into aliquots (González-Huici *et al.* 2017). One aliquot of L1 larvae was treated with 60 Gy of IR, a mutagenic agent that leads to a wide spectrum of DNA lesions including DNA double-strand breaks. The other aliquot was kept untreated to recover mutants. We selected lines that failed to propagate when subjected to IR, while propagating normally without IR treatment. IR treatment with 60 Gy did not cause a significant impairment of reproduction of the wild-type N2 strain (data not shown, Figure 1A). Here, we describe a recessive mutation (*gt2000*) that shows reduced proliferation after being irradiated at the L1 stage, to an extent similar to the previously described *tm2940* mutant in the *gen-1* Holliday junction resolvase (Bailly *et al.* 2010) (Figure 1A). *gt2000* was outcrossed three times to reduce the number of mutations caused by EMS. *gt2000* was then mapped using a combination of whole-genome sequencing and SNP mapping, which takes advantage of sequence polymorphisms between the wild-type N2 strain and a polymorphic strain initially isolated in Hawaii (Davis *et al.* 2005). The SNP mapping procedure allowed us to narrow down a ∼900 kbp region on chromosome II that was likely to contain the phenotype-causing mutation (Figure 1B). In parallel, whole-genome sequencing analysis of the mutant strain revealed a single base substitution in this genomic region, a C > T transition leading to a nonsense mutation in the *bub-3* gene (Figure 1C). *gt2000* introduces a stop codon at amino acid 104, truncating the last 239 amino acids of BUB-3. To further ascertain that *gt2000* is indeed the phenotype-causing mutation, we also analyzed the *bub-3*(*ok3437*) deletion allele provided by the Oklahoma knockout consortium (*C. elegans* Deletion Mutant Consortium 2012). We found that *bub-3*(*ok3437*) and *bub-3*(*gt2000*) L1 larvae are equally sensitive to IR (Figure 1A), further confirming that *bub-3* inactivation leads to increased IR sensitivity.

BUB-3 and MAD-3 appear to be the only components of the SAC pathway not needed for survival under physiological conditions (Nystul *et al.* 2003; Tarailo *et al.* 2007; Hajeri *et al.* 2008). To test whether the SAC pathway is generally needed for the response to DNA damage, we wondered whether mutants in *san-1*, the *C. elegans* homolog of MAD3, are equally hypersensitive to IR. We found that proliferation of *san-1*(*ok1580*) animals was equally delayed as in *bub-3* mutants upon IR treatment (Figure 1A). We generated a N-terminal GFP::BUB-3 translational fusion by genome editing in the *bub-3* genomic locus (*Materials and Methods*). This fusion protein exhibited intermediate IR hypersensitivity at 60 Gy compared to *bub-3* mutants, consistent with a compromised function of this fusion (Supplemental Material, Figure S1A). As expected, we observed GFP::BUB-3 on the metaphase plate along holocentric chromosomes, slowly fading away in anaphase (Figure S1B). When chromatin bridges induced by IR occurred, these were coated by BUB-3, consistent with *C. elegans* chromosomes being holocentric (Figure S1C). Induction of BUB-3 foci upon IR treatment was not observed (data not shown).

**Figure 2:**
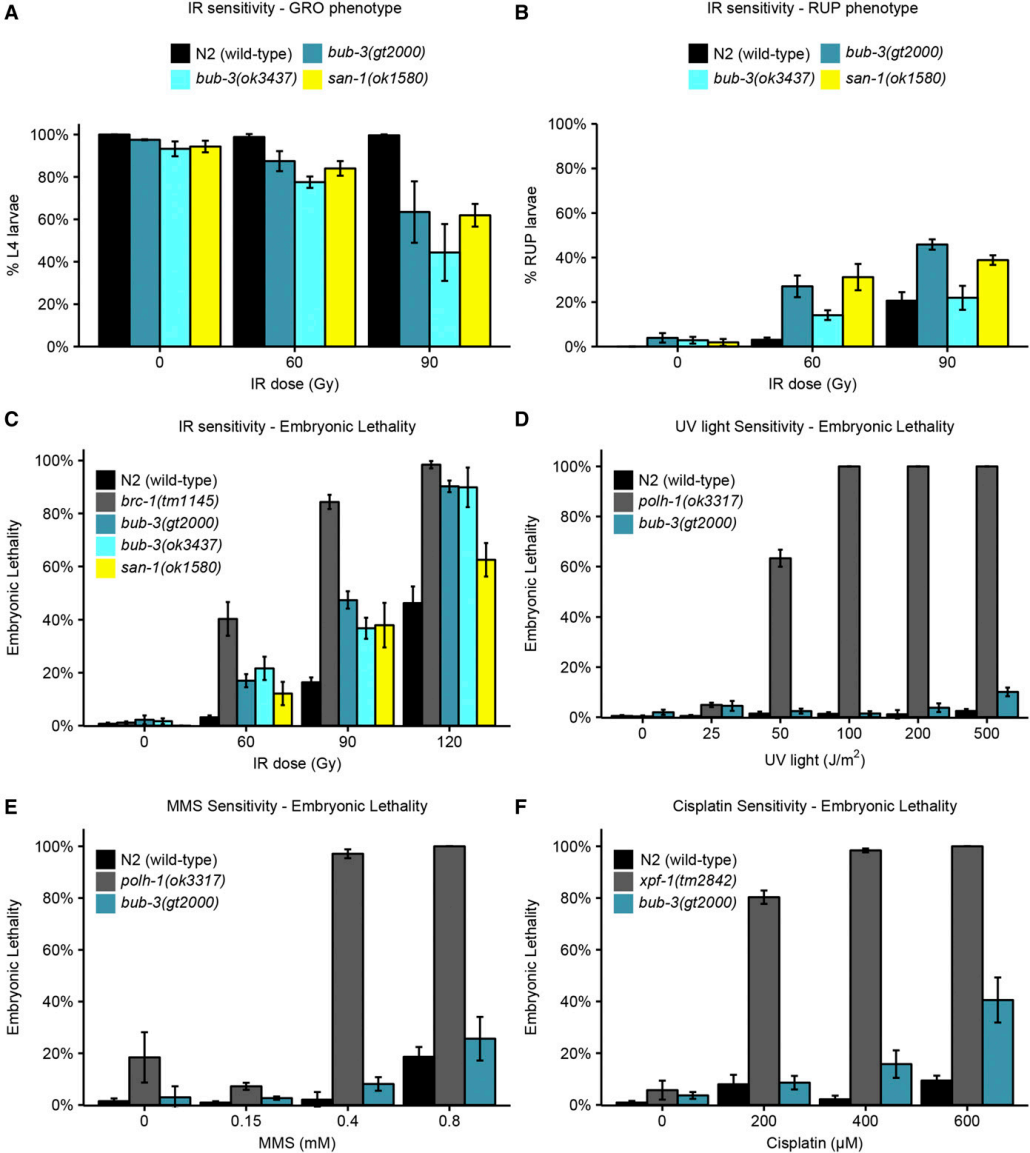
Hypersensitivity of *sac* mutants to DNA-damaging agents. (A) Quantification of the GRO phenotype in N2, *bub-3*(*gt2000*), *bub-3*(*ok3437*) and *san-1*(*ok1580*) strains treated with the indicated doses of IR. The GRO phenotype was calculated as the percentage of L1 larvae that reached the L4 stage 49 hr after irradiation. (B) Quantification of the RUP phenotype in N2, *bub-3*(*gt2000*), *bub-3*(*ok3437*), and *san-1*(*ok1580*) strains. The RUP phenotype is calculated as the percentage of animals that showed ruptured vulva 72 hr after irradiation. In (A and B) triplicates of 100 worms each were scored for each condition. (C) Embryonic lethality of N2, *bub-3*(*gt2000*), *bub-3*(*ok3437*), and *san-1*(*ok1580*) strains upon irradiation. Young adults were treated with IR at indicated doses and embryonic survival was scored as described in *Materials and Methods*. (D–F) Sensitivity of *polh-1*(*ok3317*) and *bub-3*(*gt2000*) to UV light (D) and MMS (E), and *xpf-1*(*tm2842*) and *bub-3*(*gt2000*) to cisplatin (F), measured by embryonic lethality. Error bars indicate SEM.

We next wished to determine the nature of the IR sensitivity phenotype, and to test whether *bub-3* mutants are also sensitive to other DNA-damaging agents. The impairment of proliferation upon IR treatment could be due to various defects, including a developmental delay, a high mortality of the treated animals, a reduced brood size, and increased embryonic lethality. To assay the pace of development, we irradiated L1 larvae and allowed them to grow for ∼44 hr such that 100% of wild-type N2 worms developed into the L4 stage (Figure 2A). We found that *bub-3* and *san-1* mutants displayed a moderate developmental delay (also known as the GRO phenotype) compared with wild type following irradiation (Figure 2A). Furthermore, we noticed a high incidence of ruptured mutant animals whose internal tissues extruded from the vulva, a condition that ultimately leads the animals to die prematurely (RUP phenotype, Figure 2B). Several rounds of postembryonic cell divisions are required for the proper formation of the vulva, and if the vulva does not form properly owing to cell division defects, worms rupture, with the germline protruding through the defective vulva (RUP phenotype) (O’Connell *et al.* 1998). At 90 Gy, RUP worms in *bub-3*(*ok3437*) and in wild type seem to occur at similar frequencies. However, these results are skewed by the strong GRO phenotype in *bub-3*(*ok3437*), thus allowing fewer worms of the total number plated to develop to a stage at which the ruptured phenotype becomes evident.

In addition, we found that the lethality of embryos laid ∼24 hr after irradiation of young adult stage worms was increased in *bub-3* and *san-1* mutants compared with wild type, albeit to a lesser extent than *brc-1* mutants, which are defective for homologous recombination (Figure 2C). This later sensitivity assay is known to reflect the sensitivity of meiotic germ cells, which develop into embryos 24 hr later (Gartner *et al.* 2004; Crittenden *et al.* 2006; Jaramillo-Lambert *et al.* 2007; Craig *et al.* 2012). Although the predominant type of IR-induced DNA lesions causing lethality are thought to be DNA double-strand breaks, radiation treatment can also inflict various types of secondary DNA lesions including single-strand breaks, base damage, and DNA–protein cross-links (Cadet *et al.* 2004). We thus treated wild type and *bub-3* mutants with a variety of DNA-damaging agents. UV light leads to the formation of cyclobutane pyrimidine dimers and 6,4-photoproducts. Cisplatin is a DNA cross-linking agent widely used as a chemotherapeutic agent. Besides base adducts, cisplatin forms covalent bonds linking adjacent bases (intrastrand cross-links) and bases on opposite strands (interstrand cross-links), with the former type of DNA damage occurring more frequently than the latter (Lemaire *et al.* 1991). MMS is an alkylating agent that leads to a variety of modified bases including N7-methylguanine, N3-methyladenine, and O6-methylguanine (Beranek 1990). Although MMS has long been considered a radiomimetic compound (Chlebowicz and Jachymczyk 1979; Ui *et al.* 2005), it is now widely accepted that MMS provokes formation of double-strand breaks when a replicative fork encounters alkylated bases (Paques and Haber 1999; Holway *et al.* 2006). We found that *bub-3*(*gt2000*) mutants do not show increased embryonic lethality when exposed to UV light and to MMS (Figure 2, D and E). However, *bub-3* mutants are moderately sensitive to cisplatin (Figure 2F). Our data indicate that *bub-3* mutants are not hypersensitive toward agents that predominately cause base changes. The intermediate sensitivity to cisplatin may reflect the sensitivity toward DNA cross-linking agents, or reduced DSB repair, DSBs being generated as intermediates during DNA cross-link repair (Schärer 2005).

**Figure 3:**
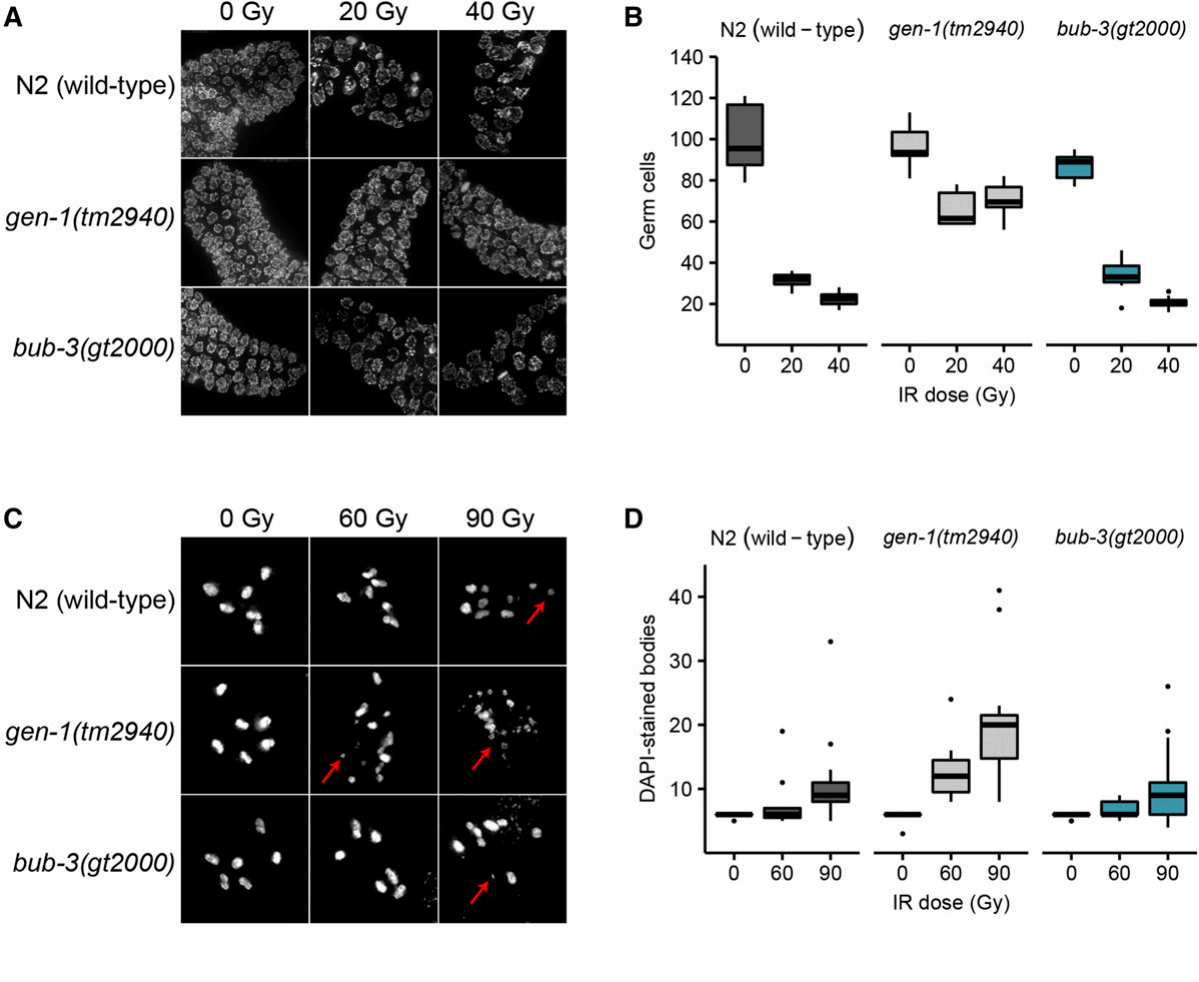
Absence of cell cycle arrest and chromosome fragmentation phenotypes in *bub-3* mutants. (A) Representative images of DAPI-stained mitotic germ cells of N2, *gen-1*(*tm2940*) and *bub-3*(*gt2000*) strains irradiated at the specified doses and DAPI stained 8 hr after irradiation of L4 staged larvae. (B) Boxplot showing the number of mitotic germ cells observed in N2, *gen-1*(*tm2940*), and *bub-3*(*gt2000*) strains 8 hr after irradiation. After image acquisition, germ cells residing in a defined volume of the distal-most region of the germline were scored. A minimum of seven germlines per IR dose were analyzed. (C) Representative images of DAPI-stained bodies in oocytes of N2, *gen-1*(*tm2940*), and *bub-3*(*gt2000*) strains irradiated with the specified doses and imaged 48 hr after IR. Red arrows highlight chromosome fragments. (D) Boxplot showing the number of DAPI-stained bodies in oocytes. A minimum of 12 oocytes per condition were analyzed.

We next wished to determine the defect that causes the radiation sensitivity of *sac* mutants. When mitotic *C. elegans* germ cells are subjected to DNA damage, a transient G2 cell cycle arrest occurs, a phenotype thought to allow for efficient repair before cells divide (Gartner *et al.* 2000; Moser *et al.* 2009). As a consequence of cell cycle arrest, the nuclei of proliferating germ cells increase their volume, as cells continue to grow without dividing (Gartner *et al.* 2000). Thus, the cell density of the mitotic region can be used as a readout for DNA damage-induced checkpoint activation (Gartner *et al.* 2000). We hypothesized that the SAC pathway might regulate DNA damage-induced cell cycle arrest. L4 larvae were irradiated and germlines were dissected 8 hr later. Germ cells residing in a given volume of the mitotic region were counted. As expected, cell density decreased proportionally to the intensity of the radiation treatment in wild type, whereas no such reduction was observed in the loss-of-function *gen-1*(*tm2940*) mutant which served as a positive control (Figure 3, A and B). GEN-1 is a Holliday junction resolvase also needed for efficient checkpoint signaling (Bailly *et al.* 2010). We could not detect a significant difference between the IR-induced cell cycle arrest in wild type and *bub-3*(*gt2000*) mutants, consistent with the notion that BUB-3 is not required for checkpoint signaling. Normal IR-induced G2 cell cycle arrest was confirmed by staining for the phosphotyrosine 15 residue of CDK-1, which is an established marker of the G2 cell cycle stage (Moser *et al.* 2009) (Figure S2). We conclude that *bub-3* does not affect DNA damage-induced G2 cell cycle arrest of mitotic germ cells.

We next wished to determine whether IR-induced DSBs persist in *bub-3* mutants. It has been shown that chromosomes at the diakinesis stage are fragmented in various DSB repair and checkpoint mutants when examined 48 hr after irradiation (Bailly *et al.* 2010; Craig *et al.* 2012). Diakinesis chromosomes are condensed and the six *C. elegans* chromosomes are readily cytologically visible in oocytes just before fertilization. As expected, we observed six DAPI-stained chromosomes in wild type, *gen*-1, and *bub-3* mutants when worms were not treated with IR (Figure 3, C and D). Chromosome fragmentation of *gen-1* became apparent upon irradiation with 60 Gy of IR as described previously (Bailly *et al.* 2010) (Figure 3, C and D). By contrast, six intact chromosomes could be observed in wild type and *bub-3*(*gt2000*) (Figure 3, C and D). Although a low level of fragmentation was evident in *bub-3* mutants upon treatment with 120 Gy of IR, this was not increased compared with wild type. (Figure 3, C and D). We next compared the kinetics of RAD-51 foci formation upon IR in wild type and *bub-3* mutants. RAD-51 is a recombinase that coats single-stranded DNA resulting from DSB processing. The number and kinetics of RAD-51 foci allows for estimating repair kinetics. Typically, 12 hr after treatment with 30 Gy ∼50% of mitotic germ cell nuclei contain repair foci, while after 16 hr foci can only be detected in a small proportion of nuclei. We found that in both wild type and *bub-3* mutant, ∼50% of nuclei contained repair foci after 12 hr, while the percentage of nuclei with RAD-51 foci dropped to ∼10% after 28 hr in both genotypes (Figure S3, A and B).

**Figure 4:**
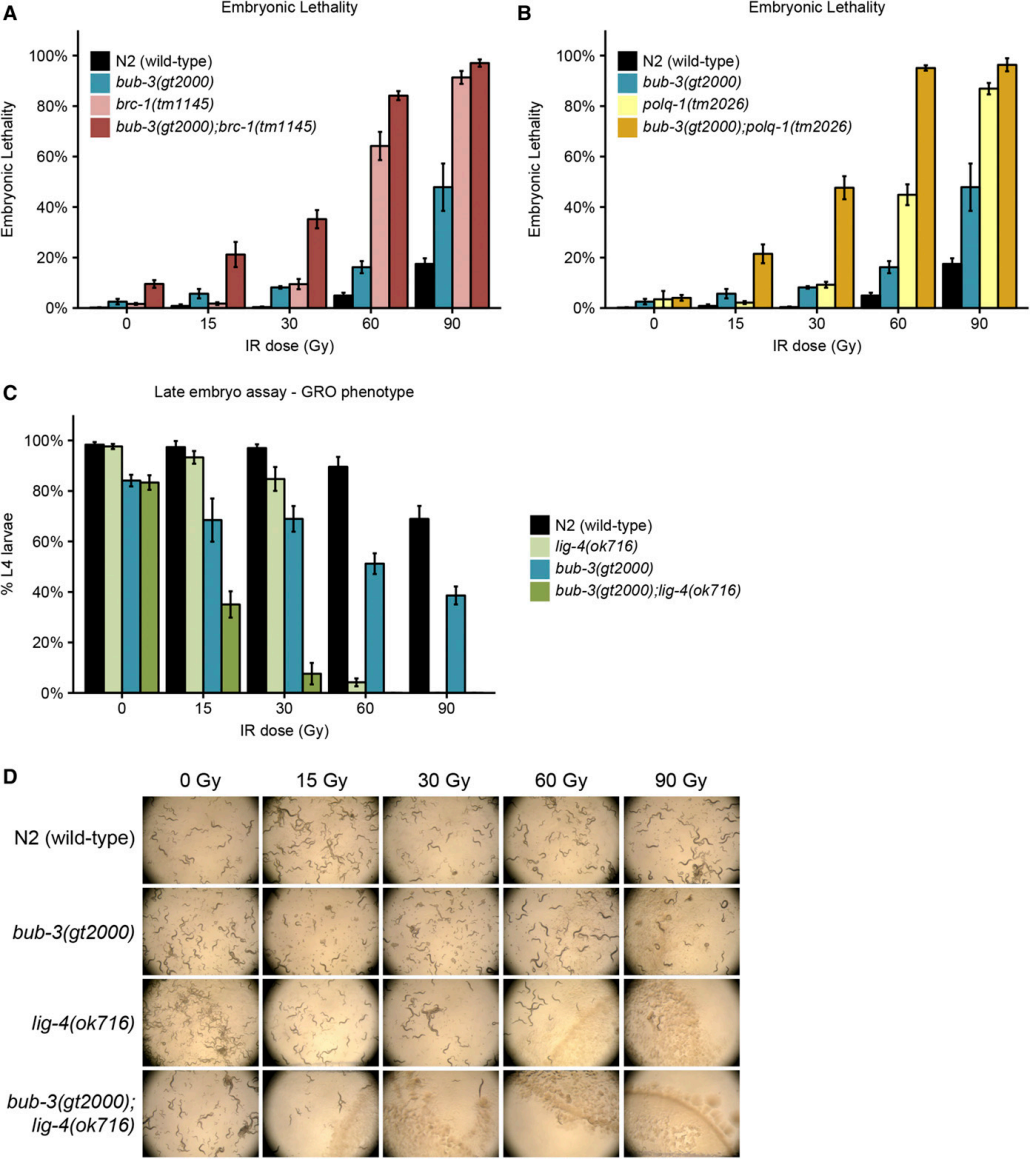
Epistasis analysis of *bub-3* with the main DSB repair pathways. (A) Embryonic lethality of *bub-3*(*gt2000*), *brc-1*(*tm1145*) single, and *bub-3*(*gt2000*);*brc-1*(*tm1145*) double mutants in response to IR. Worms were treated with IR at the young adult stage. (B) Embryonic lethality of *bub-3*(*gt2000*), *polq-1*(*tm1145*) single, and *bub3*(*gt2000*);*polq-1*(*tm1145*) double mutants in response to IR. (C) Developmental delay of N2, *bub-3*(*gt2000*), *lig-4*(*ok716*), and *bub-3*(*gt2000*); *lig-4*(*ok716*) strains upon irradiation of late-stage embryos. Late-stage embryos were irradiated and allowed to hatch and to develop. Developmental delay was quantified as the percentage of embryos that developed into L4 larvae 48 hr after irradiation. Error bars indicate SEM. (D) Representative images of NGM plates 6 d after irradiation of late-stage embryos.

Given that there is no overt change in DSB repair kinetics in *bub-3* mutants, we wondered whether BUB-3 might act together with any of the known DSB repair pathways. Repairing of DNA double-strand breaks relies at least on three major DNA repair pathways: homologous recombination (HR), nonhomologous end joining (NHEJ), and microhomology-mediated end joining (MMEJ). HR is a largely error-free DNA repair modality involving the BRCA1 protein (BRC-1 in *C. elegans*) (Boulton *et al.* 2004; Adamo *et al.* 2008). NHEJ is potentially error prone and involves the direct religation of DSBs conferred by the DNA ligase four protein (LIG-4 in *C. elegans*). In *C. elegans*, end joining is the major DSB repair modality in somatic tissues (Clejan *et al.* 2006). MMEJ is an error-prone DNA repair pathway in which blunt DNA ends are resected and scanned for microhomology recognized by polymerase θ (POLQ-1 in *C. elegans*) and used to prime DNA synthesis to fill the gaps (Roerink *et al.* 2014; van Schendel *et al.* 2016). We thus generated *bub-3* double mutants with *brc-1*, *lig-4*, and *polq-1*, known to be required for HR, NHEJ, and MMEJ, respectively. We analyzed single and double mutants by irradiating young adults and quantifying the extent of embryonic lethality. As previously reported, *brc-1* and *polq-1* single mutants were hypersensitive to IR (Figure 4, A and B) (Boulton *et al.* 2004; Muzzini *et al.* 2008). Interestingly, both *bub-3*;*polq-1* and *bub-3*;*brc-1* double mutants were more sensitive to IR compared with the respective single mutants (Figure 4, A and B), consistent with *bub-3* functioning in parallel to HR and MMEJ. Given that it acts predominantly in somatic cells, NHEJ is commonly assayed by measuring the growth delay of irradiated late-stage embryos. Scoring the percentage of embryos reaching the L4 stage ∼48 hr after irradiation, we found that both single mutants showed retarded development, the phenotype being stronger in *lig-4* mutants upon treatment with high doses of irradiation. The growth delay of the *bub-3*;*lig-4* double mutant was dramatically increased, consistent with a role of BUB-3 in somatic tissues in parallel to NHEJ (Figure 4, C and D). In summary, we provide genetic evidence that the SAC pathway acts independently of the known DSB repair pathways.

**Figure 5:**
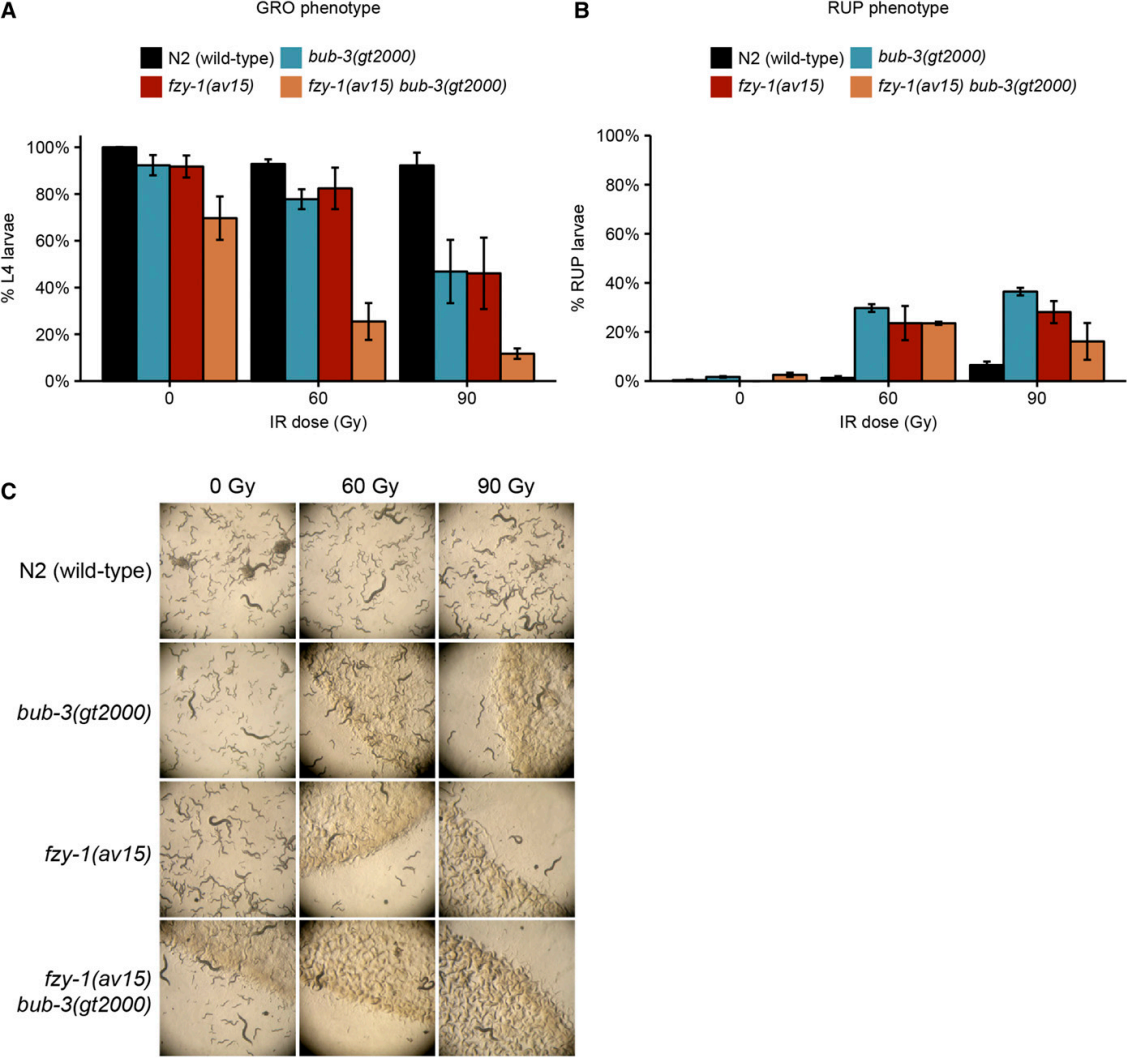
*fzy-1*(*av15*) mutants are hypersensitive to IR. (A) Quantification of the GRO phenotype in N2, *bub-3*(*gt2000*), *fzy-1*(*av15*) single, and *fzy-1*(*av15*) *bub-3*(*gt2000*) double mutants after irradiation of L1 larvae at the indicated doses. (B) Quantification of the RUP phenotype in N2, *bub-3*(*gt2000*), *fzy-1*(*av15*) single, and *fzy-1*(*av15*) *bub-3*(*gt2000*) double mutants after irradiation of L1 larvae at the indicated doses. Error bars indicate SEM. (C) Representative images of NGM plates 5 d after irradiation of late-stage embryos.

It is established that the SAC delays progression to anaphase by inhibiting the Cdc20/FZY-1 activator of the APC (Hwang *et al.* 1998). We considered the possibility that precocious entry into M-phase in *bub-3* and *mad-3* mutants might contribute to the increased sensitivity toward IR. A CDC-20 gain-of-function allele, *fzy-1*(*av15*), which leads to precocious M-phase entry, is available (Stein *et al.* 2007; Lawrence *et al.* 2015). Consistent with the role of precocious M-phase entry playing a part in conferring IR sensitivity, we found that treatment of both *bub-3* and *fzy-1*(*av15*) mutants led to a heightened sensitivity to IR, based on developmental delay phenotypes and the increased incidence of the ruptured vulva phenotype (Figure 5, A–C). We observed an even stronger phenotype when both mutants were combined. We investigated whether treatment of *bub-3* mutants leads to precocious cell cycle progression in one- and two-cell stage embryos, but could not find evidence for this (Figure S4). However, it is known that checkpoint regulation is weak in rapidly dividing *C. elegans* embryonic cells (Holway *et al.* 2006), and we thus assume that a change in cell cycle timing might occur during later cell divisions, leading to the slow growth and rupture phenotypes.

## Discussion

In this study, we isolated a mutation (*gt2000*) from a forward genetic screen that confers hypersensitivity to IR. We found that *gt2000* leads to a premature stop codon in *bub-3*, and that the *bub-3*(*ok3437*) deletion allele similarly confers hypersensitivity to IR. Irradiation of *bub-3*(*gt2000*) L1 larvae induces development defects such as a developmental delay and a ruptured vulva phenotype. Moreover, irradiation of *bub-3*(*gt2000*) young adults increases the lethality in embryos derived from those animals. *san-1*(*ok1580*) mutants are also hypersensitive to IR, consistent with the notion that the SAC pathway might be activated when DNA damage is inflicted. Treatment with a panel of DNA-damaging agents indicates that the SAC pathway might be required to mend DSBs, while not being required for the repair of damaged DNA bases.

SAC components BUB-3 and SAN-1 are not essential for viability under unperturbed growth conditions, and the corresponding mutants do not show an overt developmental phenotype (Nystul *et al.* 2003; Tarailo *et al.* 2007; Hajeri *et al.* 2008, and our data). The SAC is composed of two branches both contributing to APC inactivation by CDC20 binding (Essex *et al.* 2009). The Mad2 conformational change needed for Cdc20 binding and inhibition is facilitated by the C-Mad2:Mad1 complex linked to unattached kinetochores. By contrast, Bub3 interacts with Mad3 to then bind to the inhibitory C-Mad2:Cdc20 complex (Essex *et al.* 2009). Components of this latter pathway are not needed for viability. The stronger phenotype observed in the *fzy-1*(*av15*) *bub-3*(*gt2000*) double mutant compared with the two single mutants may be explained by the fact that *fzy-1*(*av15*) is a gain-of-function allele; gain of function being ascribed to reduced MAD-2 binding to FZY-1/CDC20, thus causing precocious cell cycle progression (Stein *et al.* 2007; Lawrence *et al.* 2015). The *mad-2* deletion phenotype is stronger than the *bub-3* phenotype, the former leading to lethality, while overt *bub-3* phenotypes are only evident upon treatment with agents such as IR. Thus, precocious cell cycle progression might be stronger when *fzy-1*(*av15*) and a *bub-3*(*null*) allele are combined.

Our data are consistent with the SAC acting in response to DNA damage during both germ cell development and somatic development. DSB repair is predominantly ascribed to HR and MMEJ in the germline, while NHEJ acts in somatic cells. Our double mutant analysis indicates that BUB-3 might act independently of the HR, MMEJ, and NHEJ pathways. It remains to be determined how BUB-3 and SAN-1/MAD-3 prevent hypersensitivity to IR. It is possible that these two proteins directly act as DSB repair factors. Consistently, previous findings showed that MAD-2 colocalizes with RAD-51 foci at the nuclear periphery in a DDR-dependent fashion (Lawrence *et al.* 2015). Moreover, lack of MAD-1, MAD-2, SAN-1/MAD-3, and BUB-3 renders *C. elegans* mitotically dividing germ cells unable to process DNA damage efficiently (Lawrence *et al.* 2015). We could not observe any difference between wild type and *bub-3* mutants in the number of RAD-51 foci and IR-induced chromosome fragments. It was shown that BUB-3 affects repair of hydroxyurea-induced DNA damage to a lesser extent than the MAD proteins (Lawrence *et al.* 2015). Thus, our data are compatible with *bub-3* not affecting the kinetics of RAD-51 unloading. It has been previously shown in yeast that the function of the kinetochore is perturbed when double-strand breaks are induced in close proximity, leading to SAC activation (Dotiwala *et al.* 2010). Given the holocentric nature of *C. elegans* chromosomes, we cannot rule out this possibility. Finally, we entertain the possibility that the BUB-3 and SAN-1 branch of the SAC pathway may confer hypersensitivity to IR by causing precocious entry into mitosis. This would be consistent with the IR sensitivity of the *fzy-1*(*av15*) gain-of-function allele previously shown to advance entry into mitosis. We investigated this hypothesis in early embryos, a system amenable to precisely measuring cell cycle timing. While we observed that cell cycle timing is extended when embryos are treated with IR, no difference between wild-type and *bub-3* mutant embryos could be detected. Nevertheless, checkpoint phenotypes tend to be very weak during early embryogenesis (Holway *et al.* 2006), and we thus postulate that precocious entry into mitosis during development could contribute to the IR sensitivity of *bub-3* and *san-1* mutants. In summary, we found that *C. elegans bub-3* and *san-1* mutants are hypersensitive to IR.

## Supplementary Material

Supplemental material is available online at www.g3journal.org/lookup/suppl/doi:10.1534/g3.117.1122/-/DC1.
