# Supplementary figures and images for: Caenorhabditis elegans BUB-3 and SAN-1/MAD3 Spindle Assembly Checkpoint Components Are Required for Genome Stability in Response to Treatment with Ionizing Radiation
Source: G3 (Bethesda). 2017 Oct 18;7(12):3875–85. doi: 10.1534/g3.117.1122 (PMC5714485; doi:10.1534/g3.117.1122)

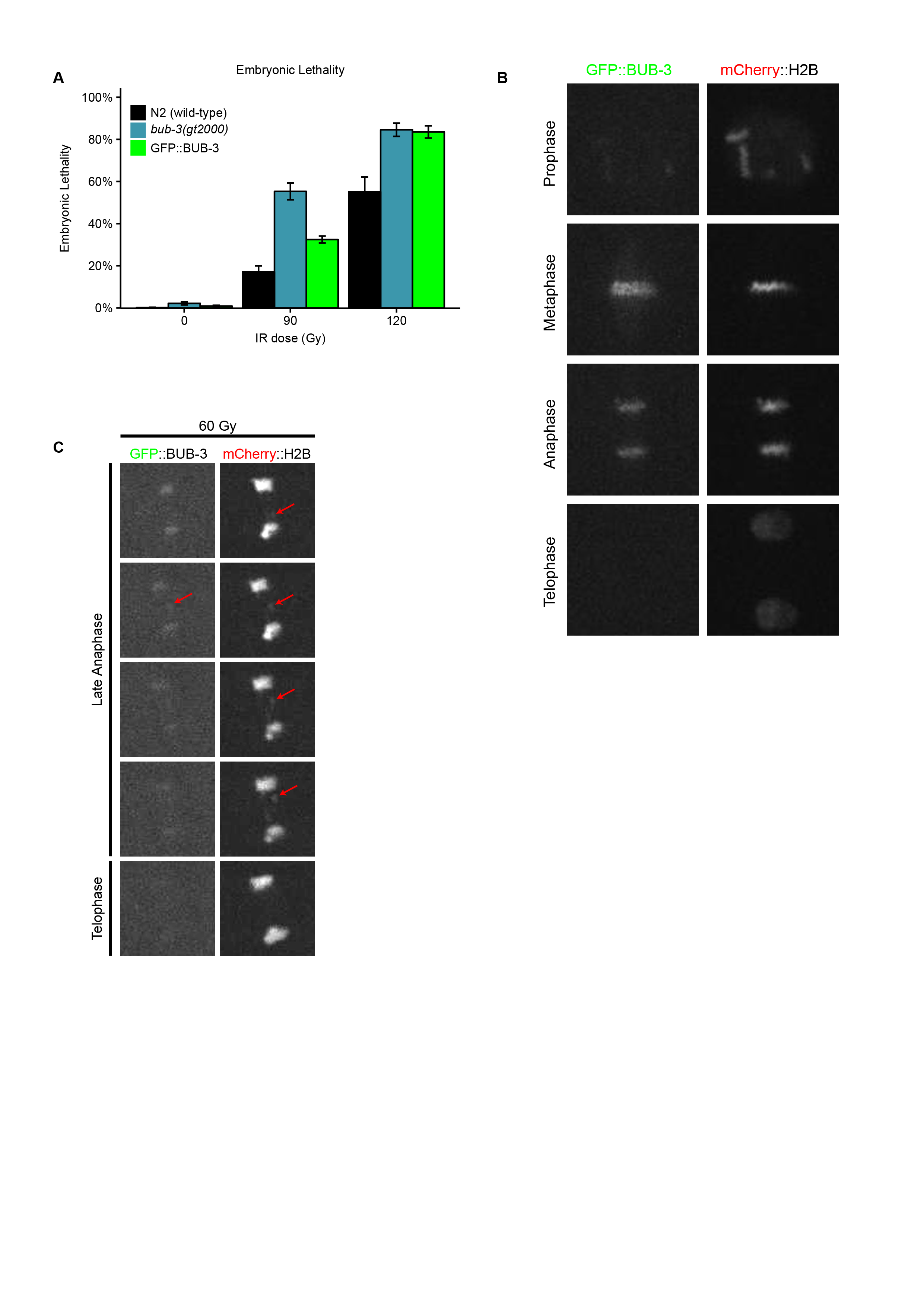

Supplement: Supplementary file 1 [file 3875FigureS1.tif]

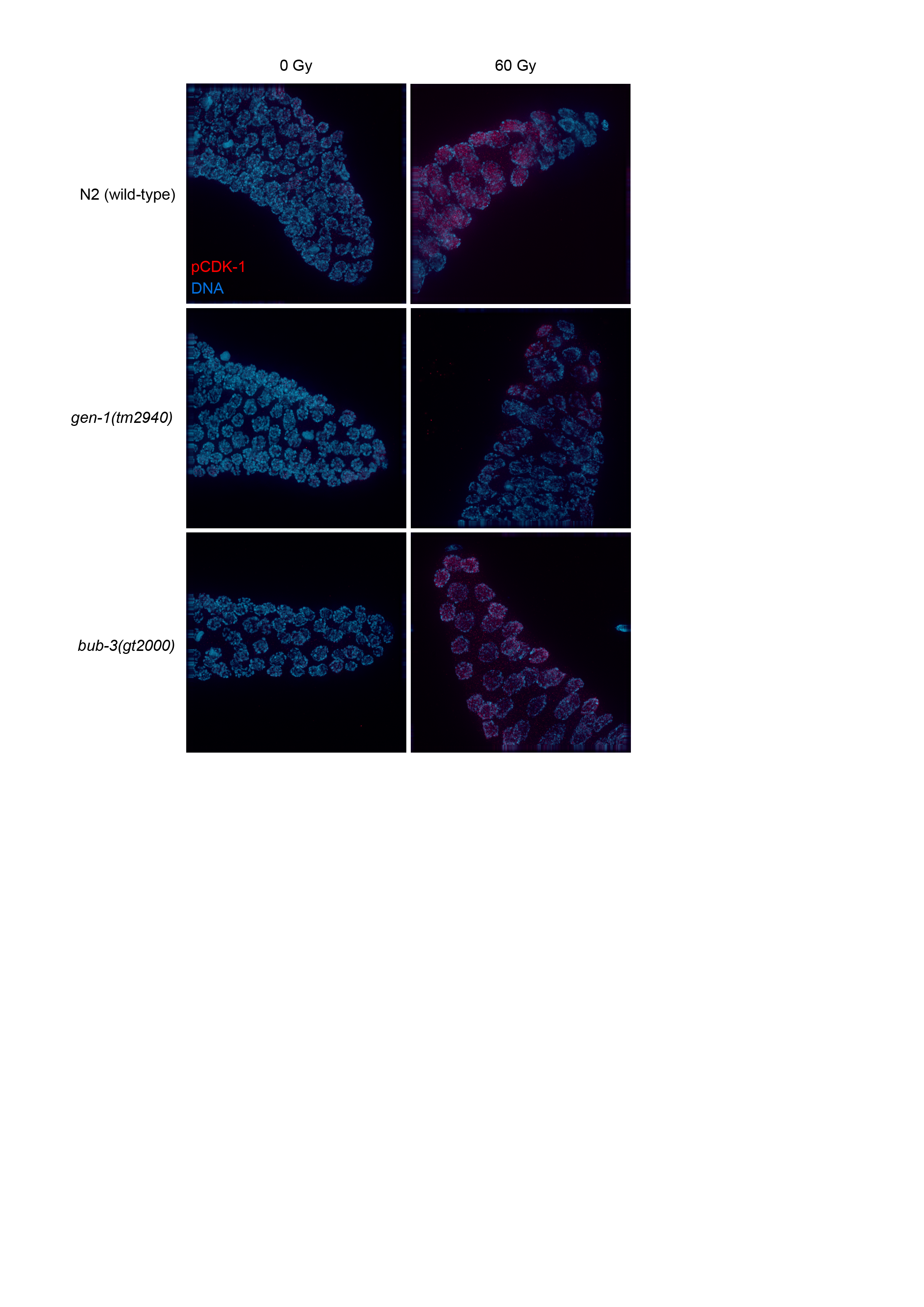

Supplement: Supplementary file 2 [file 3875FigureS2.tif]

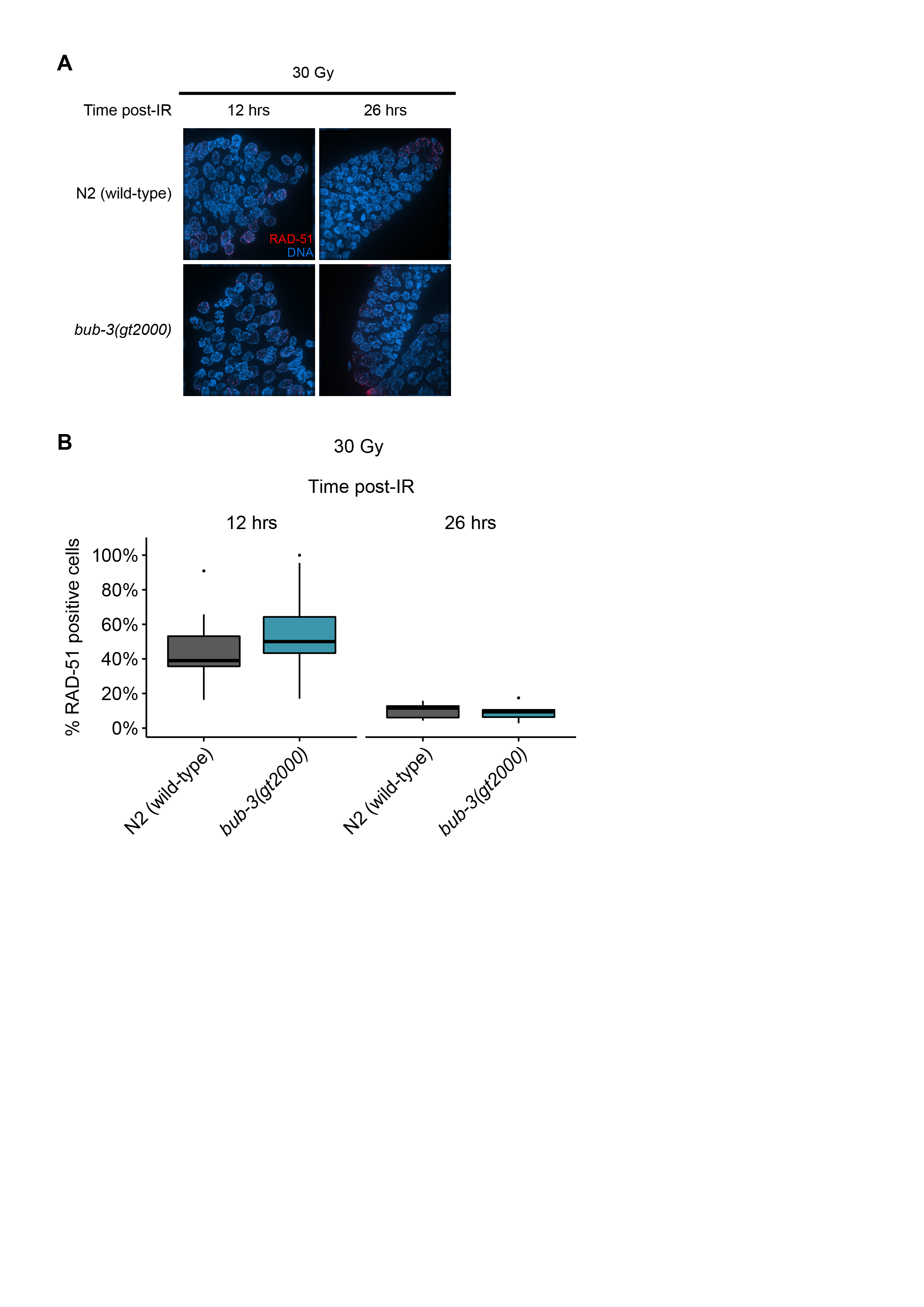

Supplement: Supplementary file 3 [file 3875FigureS3.tif]

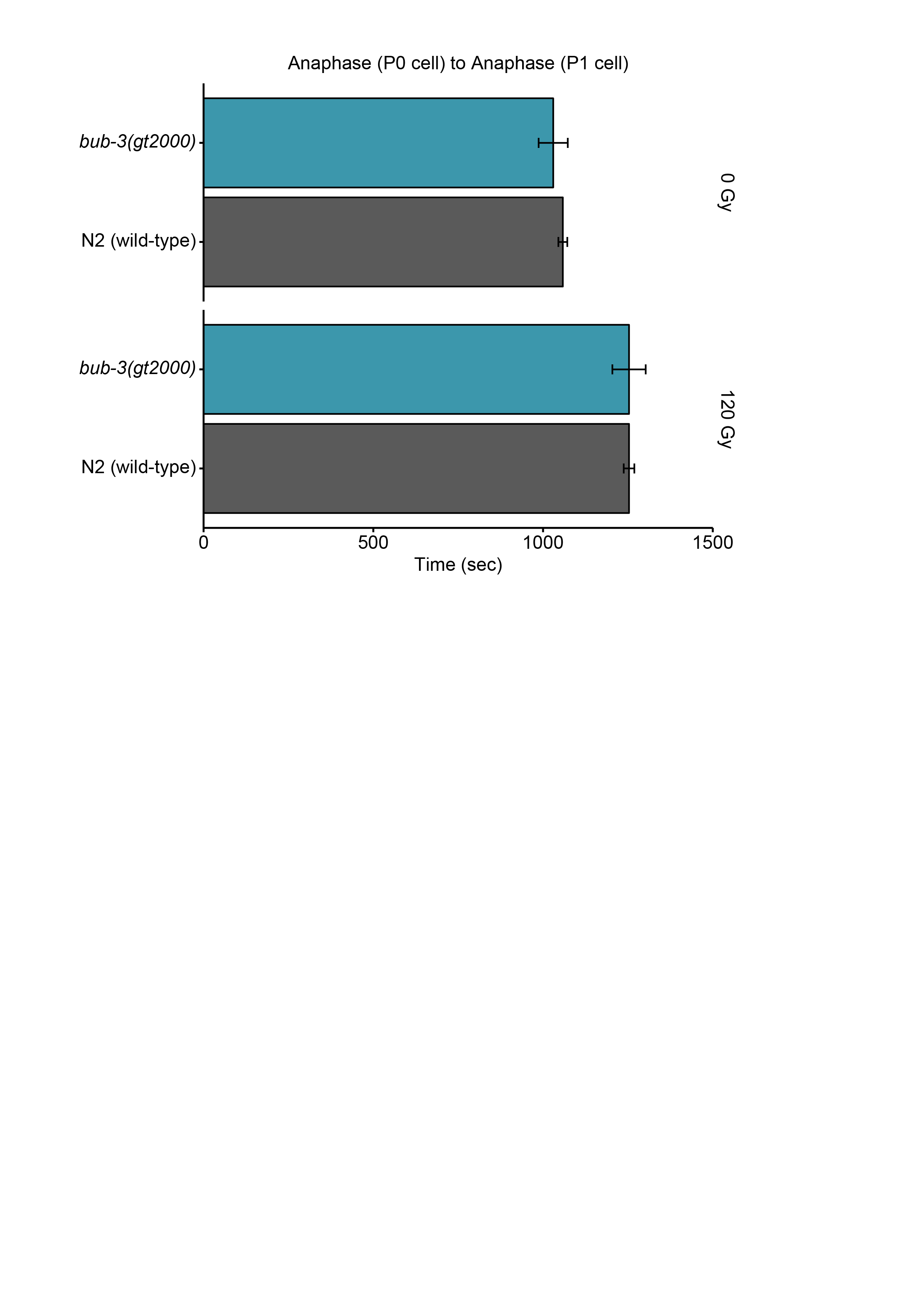

Supplement: Supplementary file 4 [file 3875FigureS4.tif]
