# Supplementary material for: Caenorhabditis elegans BUB-3 and SAN-1/MAD3 Spindle Assembly Checkpoint Components Are Required for Genome Stability in Response to Treatment with Ionizing Radiation
Source: G3 (Bethesda). 2017 Oct 18;7(12):3875–85. doi: 10.1534/g3.117.1122 (PMC5714485; doi:10.1534/g3.117.1122)
Supplement: Supplementary file 5 [file 3875FileS1.docx]

**FIGURE S1. Embryonic lethality of irradiated GFP::BUB-3 strain and BUB-3 localization in one-cell embryo.** (A) Embryonic lethality of N2, *bub-3(gt2000)*, GFP::BUB-3 strains after irradiation at the specified doses. (B) GFP::BUB-3 and mCherry::H2B localization in one-cell embryo at mitotic prophase, metaphase, anaphase and telophase. (C) Time-lapse images of an irradiated one-cell embryo showing GFP::BUB-3 localization on lagging chromosome during late anaphase/early telophase.

**FIGURE S2.** **PhosphoCDK-1^Tyr15^ immunostaining.** PhosphoCDK-1^Tyr15^ immunostaining of N2, *gen-1(tm2940)* and *bub-3(gt2000)* germlines (mitotic region) 8 hr after irradiation of L4 larvae.

**FIGURE S3.** **RAD-51 immunostaining.** (A) RAD-51 immunostaining in N2 and *bub-3(gt2000)* germlines (mitotic region) 12 hr and 26 hr post-irradiation with 30 Gy. (B) Boxplot showing the percentage of RAD-51 positive mitotic germ cells in N2 and *bub-3(gt2000)* strains 12 and 26 hr after irradiation of young adults with 30 Gy. n = 13, 9, 23, 6 germlines analyzed for N2 (12 hr), N2 (26 hr), *bub-3(gt2000)* (12 hr) and *bub-3(gt2000)* (26 hr), respectively.

**FIGURE S4.** **Analysis of cell cycle timing in early embryos.** Barplot showing the time taken from anaphase onset in P0 (one-cell embryo) to anaphase onset in the P1 cell (two-cell embryo) in N2 and *bub-3(gt2000)* without irradiation and following irradiation with 120 Gy. n = 6, 6, 6, 3 germlines analyzed for N2 (0 Gy), N2 (120 Gy), *bub-3(gt2000)* (0 Gy), *bub-3(gt2000)* (120 Gy), respectively. Error bars indicate SEM.
